# Evaluation of axial length to identify the effects of monocular 0.125% atropine treatment for pediatric anisometropia

**DOI:** 10.1038/s41598-021-96414-4

**Published:** 2021-11-02

**Authors:** Po-Hsiang Kao, Lan-Hsin Chuang, Chi-Chun Lai, Shin-Yi Chen, Ken-Kuo Lin, Jiahn-Shing Lee, Chiun-Ho Hou, Chueh-Tan Chen, Yu-Kai Kuo, Chi-Chin Sun, Chun-Fu Liu

**Affiliations:** 1 Department of Medicine, Chang Gung Memorial Hospital, Linkou, Taiwan; 2 Department of Ophthalmology, Chang Gung Memorial Hospital, Keelung, Taiwan; 3 College of Medicine, Chang Gung University, Taoyuan, Taiwan; 4 Department of Ophthalmology, Chang Gung Memorial Hospital, Linkou, Taiwan; 5 Institute of Traditional Medicine, National Yang Ming Chiao Tung University, Taipei, Taiwan; 6 Program in Molecular Medicine, National Yang Ming Chiao Tung University, Taipei, Taiwan

**Keywords:** Outcomes research, Paediatric research, Eye diseases

## Abstract

The aim of the study is to determine the effects of monocular 0.125% atropine daily treatment on the longer axial length (AL) eyes in children with pediatric anisometropia. This was a retrospective cohort study. The charts of children with anisometropia (aged 6–15 years) who had a > 0.2-mm difference in AL between the two eyes were reviewed. Children who received monocular treatment of 0.125% atropine in the eye with longer AL were included for final analysis. The main outcome measure was the difference in AL between the two eyes after treatment. Regression analysis was used to model the changes in AL according to the time of treatment in both eyes. Finally, forty eyes in 20 patients (mean age 10.2 years) were included in the analyses. During the treatment period, AL was controlled in the treated eyes (*p* = 0.389) but elongated significantly in the untreated eyes (*p* < 0.001). The difference in AL between the treated and untreated eyes decreased from 0.57 to 0.22 mm (*p* < 0.001) after the 1-year treatment period. In the regression model, the best fit for the relationship between changes in AL and time during the treatment period in the treated eyes was the quadratic regression model with a concave function. In conclusion, these data suggest that 0.125% atropine daily is an effective treatment to reduce the interocular difference of AL in eyes with axial anisometropia. This pilot study provides useful information for future prospective and larger studies of atropine for the treatment of pediatric axial anisometropia.

## Introduction

Anisometropia is a condition in which the two eyes have unequal refractive error and is commonly defined as a difference in spherical equivalent refractive error (SEQ) > 1.0 diopter (D) between eyes^1–3^. The prevalence of anisometropia is about 4–7% in Norway, the USA, and China^1,3,4^ and is about 5.3% among young schoolchildren in Taiwan^2^. Anisometropia can lead to the development of amblyopia, but even in the absence of amblyopia, people with anisometropia may experience visual fatigue syndrome, subnormal binocular function, and reduced stereopsis^5^.

Anisometropia can be classified according to the SEQ in both eyes into simple/compound hyperopic or myopic and mixed anisometropia^5,6^. A variety of treatments for anisometropia have been reported, but there is no consensus on the best treatment^5,7^. Because most cases of anisometropia result from a difference in axial length (AL)^5^, measuring this length may be useful for the direct monitoring of axial anisometropia, a method that is similar to the myopia control strategy^8^.

Atropine is thought to retard AL elongation in myopic eyes, and some studies have reported that atropine reduces the progression of axial myopia^8,9^. Daily use of lower-dose atropine (< 0.125%) is the most common treatment for myopia because this dose is less likely to cause adverse and rebound effects while remaining adequate for the treatment of myopia^8,10^. Daily use of lower-dose atropine in the eye with longer AL may be a rational method to correct axial anisometropia.

A previous study reported that monocular administration of 1% atropine every 3 days in the eye with the longer AL was an effective treatment for myopic and mixed-types of anisometropia^5^. However, the most common practice involves daily use of lower-dose atropine for treating myopia in school students^8^. No studies have reported on the effects of lower-dose atropine on pediatric axial anisometropia. Therefore, the current study was conducted to examine the effects of daily treatment of monocular 0.125% atropine for axial anisometropia in children.

## Methods

### Participants

This retrospective cohort study was conducted at the Keelung and Taipei branches of the Chang Gung Memorial Hospital, Taiwan. The medical charts of children aged 6–15 years who had received daily treatment of monocular 0.125% atropine in the eye with the longer AL for anisometropia correction were reviewed. The records were obtained from the Atropine Treatment Continuous Follow-up open database, which contains the complete medical records and detailed examination results of patients who had received atropine treatment at the Keelung and Taipei branches of the Chang Gung Memorial Hospital from February 2017. The study and a waiver for consent to participation were approved by the Institutional Review Board of Chang Gung Memorial Hospital (Approval No. 202001771B0) and followed the tenets of the Declaration of Helsinki.

### Inclusion and exclusion criteria

A difference in interocular AL > 0.2 mm was defined as “axial anisometropia,” and patients with this difference were included in the current study. The exclusion criteria were as follows at the time point of initiating treatment: (1) previous treatment with atropine in either eye; (2) history of amblyopia in either eye; (3) astigmatism > 3.50 D in either eye; (4) hyperopia with SEQ > 0.75 D in the longer-AL eye; (5) SEQ < − 3.00 D in either eye; (6) history of other ocular pathology except for anisometropia in either eye; or (7) compliance of < 80% with the atropine treatment during the follow-up.

### Ocular examination and measurement of AL

The best-corrected visual acuity before cycloplegia and the objective refractive error after cycloplegia were measured at the first visit. The latter was obtained 1 h after the first instillation of 1% tropicamide (Mydriacyl; Alcon Vision, LLC, Fort Worth, TX, USA) plus 10% phenylephrine hydrochloride (Phenylephrine Eye Drops; Wu Fu Laboratories Co. Ltd., Yilan, Taiwan). In detail, for cycloplegia induction, both drops were instilled together every 10 min three times. Pupil enlargement with no light reflex was confirmed before measurement of the objective refractive error after cycloplegia using an Auto Ref/Keratometer (ARK-1a/ARK-1; Nidek Co., Ltd., Gamagori, Japan). The mean value of three consecutive measurements was calculated for the final analysis.

Some records lacked the cycloplegic refractive error values at the time points of initiating and stopping monocular atropine treatments, so we calculated these values based on the cycloplegic refractive error at the baseline and the changes in AL. For this calculation, a 0.1 mm AL elongation was presumed with a change of –0.25 D in SEQ^11^.

AL was measured by experienced technicians using an IOLMaster 500 optical measurement device (Carl Zeiss Meditec AG, Jena, Germany) in the Keelung branch or an AL SCAN device (Nidek Co., Ltd, Gamagori, Japan) in the Taipei branch. Annual changes in AL were calculated as [(the difference between the last and the first AL, in mm) / (the number of days between the last and the first AL measurement)] × 365.25 for further analysis.

### Monocular atropine treatment protocol

As per the current strategy for myopia monitoring in school-aged children, we recorded the children’s baseline cycloplegic refractive error and AL at their first visit and planned to follow up with them at 3-month intervals. We used a similar protocol to follow up with these children with anisometropia because the eye with the longer AL has a tendency toward myopia progression. If the eye with the longer AL elongated slower compared with that of children of the same age, we continued observing them until the annual AL change accelerated to that within the treatment range^8,12^. If the eye with the longer AL elongated rapidly at the next follow-up or tended to progress to high myopia (< − 6 D), the child was given monocular 0.125% atropine treatment immediately^8^. We then followed up with the children at 3-month intervals thereafter. When the AL of the untreated eye reached that of the other eye, monocular therapy was stopped and both eyes were treated to control myopia.

During the treatment period, all children were treated with 0.125% atropine sulfate (Tropine Eye Drops; Aseptic Innovative Medicine Co., Ltd., Taoyuan, Taiwan). They were instructed to instill one drop into the eye with the longer AL before going to sleep every night. Compliance was assessed by carefully asking the patients and their parents at each follow-up visit. The pupil size was also used to check the reported compliance.

### Statistical analysis

All statistical analyses were conducted using IBM SPSS Statistics for Windows (version 25.0; IBM Corp., Armonk, NY, USA). AL and treatment parameters were compared between the treated and untreated eyes at the first visit and before and after treatment time using the paired-sample *t* test. Changes in AL in the treated and untreated eyes between the three time points were analyzed using generalized estimating equations (GEEs). The link function was that the identity and distribution were normal in the GEE. Exchangeable working correlation and robust standard error were adopted to identify the significance of parameters with the lowest Corrected Quasi-likelihood under the Independence Model Criterion (QICC). Changes in AL during the observation and treatment periods were also analyzed using regression analysis to identify the best-fitting regression models (linear or quadratic using adjusted *R*^2^). A two-tailed *p* value < 0.05 was accepted as significant; no adjustments of the alpha error were made in this study. According to the study by Lin et al.^5^, the interocular differences in axial length before and after treatment were 0.74 ± 0.32 mm and 0.29 ± 0.32 mm, respectively. Given the reported effect size (Cohen’s d = 1.41), a type I error rate of 5%, and a power of 95% (1 − type II error), the required minimum sample size was calculated as 30 eyes.

## Results

We initially reviewed 73 charts of children with axial anisometropia. We excluded 53 children at the time point of initiating treatment for the following reasons: 18 had received prior atropine treatment in either eye, seven had a history of amblyopia in either eye, five had astigmatism > 3.50 D in either eye, five had hyperopia with SEQ > 0.75 D in longer-AL eye, two had SEQ < − 3.00 D in either eye, two had another type of pathology in either eye, and 14 had compliance of < 80% with the atropine treatment during the follow-up.

Ultimately, 20 children were included in the final analysis. The detailed demographic and treatment data are summarized in Supplementary Tables 1 and 2, respectively. The mean age was 10.2 years and they were 11 boys and 9 girls. Nine children eventually received atropine treatments in both eyes because the refractive error indicated development of myopia and similar AL in both eyes were found. The mean treatment duration for these nine children was 14.2 months, and their data after atropine treatment of both eyes were excluded from the analyses.

### Differences in treatment parameters during the observation and treatment periods between the treated and untreated eyes

The AL differences between treated and untreated eyes at first visit, before and after treatment time points, are summarized in Table 1. There are significant differences of SEQ and AL at all time points between treated and untreated eyes. Furthermore, during the observation period, the changes in AL were significantly larger in the later-treated eyes than in the untreated eyes; however, during the treatment period, the result was inverse. The mean observation duration was 10.7 ± 10.5 months and the mean treatment duration was 13.7 ± 9.6 months (mean ± SD). Data were available for 16 patients during the observation period, and the other four patients (cases 1, 2, 7, and 18) were treated immediately at their first visit because of strong requests for myopia control and anisometropia treatment by their parents.

**Table 1:** Differences in treatment parameters between treated and untreated eyes at the three time points and annual changes in AL during the observation and treatment periods.

Parameter	Treated eye (*N* = 20)	Untreated eye (*N* = 20)	*p* value*
SEQ at first visit (D)	− 0.55 ± 1.11	0.67 ± 1.14	< 0.001
AL at first visit (mm)	23.63 ± 1.21	23.16 ± 1.04	< 0.001
SEQ before treatment (D)	–1.25 ± 0.94	0.15 ± 1.29	< 0.001
AL before treatment (mm)	23.91 ± 1.02	23.35 ± 0.92	< 0.001
SEQ after treatment (D)	− 1.39 ± 1.16	–0.67 ± 1.29	0.004
AL after treatment (mm)	23.97 ± 1.06	23.70 ± 1.01	0.005
**Annual changes in AL (mm)**			
Observation period (mean = 10.7 months, median = 4.7 months, *N* = 16)	0.40 ± 0.26	0.21 ± 0.140	0.008
Treatment period (mean = 13.7 months, median = 11.6 months)	− 0.03 ± 0.21	0.32 ± 0.24	< 0.001

*p < 0.05, paired *t* test.

Continuous data are expressed as mean ± standard deviation.

SEQ, spherical equivalent refractive error; AL, axial length; *N*, number of eyes.

### Changes in AL in the treated and untreated eyes and changes in interocular differences after treatment

**Figure 1:**
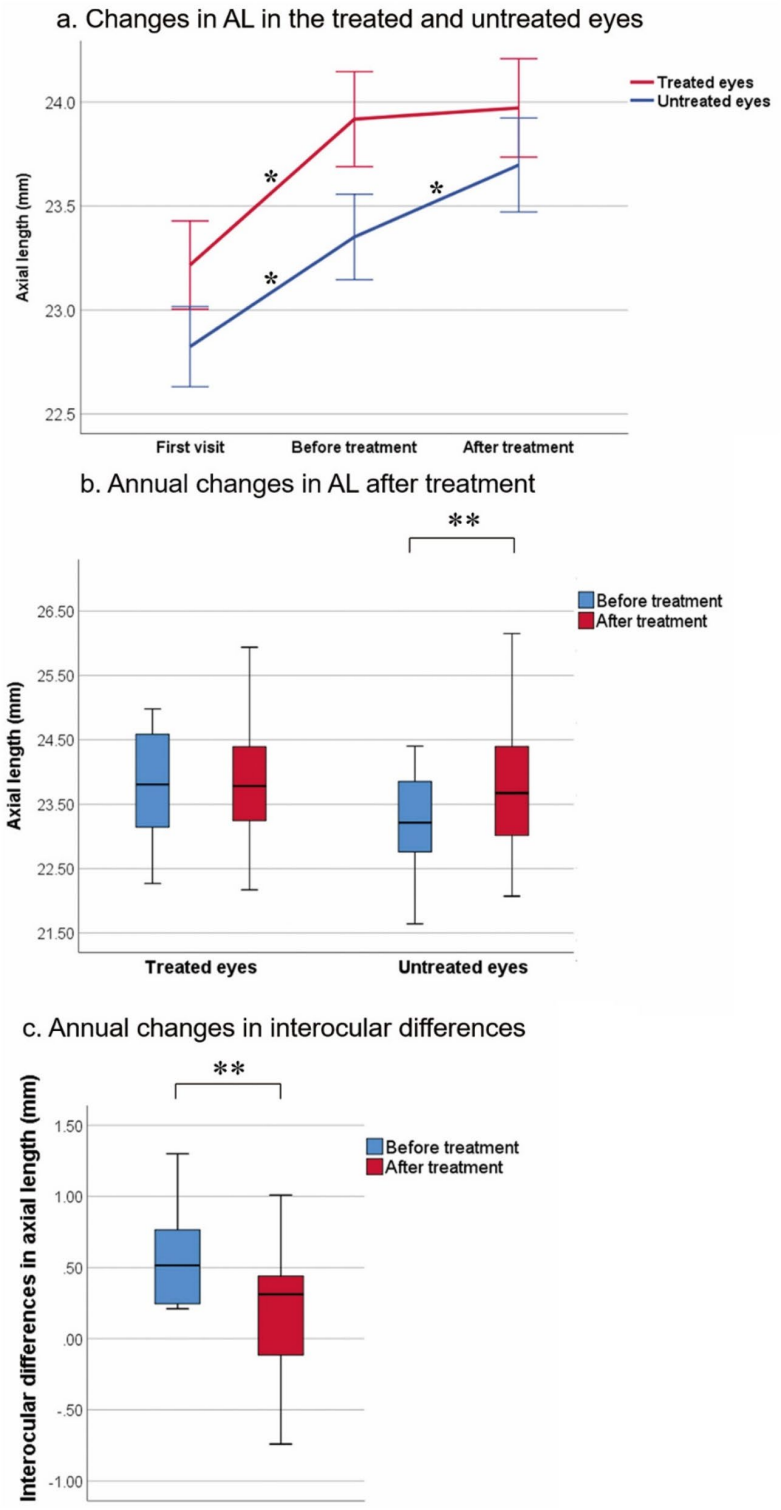
Changes in AL in the treated and untreated eyes during the observation (*n* = 32) and treatment (*n* = 40) periods and changes in interocular differences after treatment. (**a**) Changes in AL in the treated and untreated eyes. In the treated eyes, AL changed significantly during the observation period (*p* < 0.001), but not during the treatment period (*p* = 0.389). In the untreated eyes, AL changed significantly during both the observation and treatment periods (both *p* < 0.001). (**b**) Annual changes in AL were not significant in the treated eyes (*p* = 0.596), but were significant in the untreated eyes (*p* < 0.001). (**c**) Annual changes in interocular differences decreased significantly (*p* < 0.001). *p < 0.001, generalized estimating equation; **p < 0.001, paired-sample *t* test. Error bars in panel (**a**) indicate one standard error at each time point and for each group; box plots in panels (**b**) and (**c**) indicate the minimum, first quartile, median, third quartile, and maximum values.

The changes in AL and annual changes in AL in the treated and untreated eyes are summarized in Fig. 1a and b, respectively; The annual changes in interocular differences after treatment are shown in Fig. 1c.

In the treated eye, AL differed significantly from before to after the observation period (*p* < 0.001) but did not change during the treatment period (*p* = 0.389). In the untreated eye, AL differed significantly from before to after both the observation and treatment periods (both *p* < 0.001) (Fig. 1a).

After adjustment for the 1-year treatment period, AL remained unchanged in the treated eye (*p* = 0.596) but was significantly longer in the untreated eye (*p* < 0.001) (Fig. 1b).

The mean interocular difference in AL changed from 0.57 to 0.22 mm after treatment for 1 year, the difference decreased significantly (*p* < 0.001) (Fig. 1c).

### Regression models to identify changes in AL in the treated and untreated eyes during the observation and treatment periods

**Figure 2:**
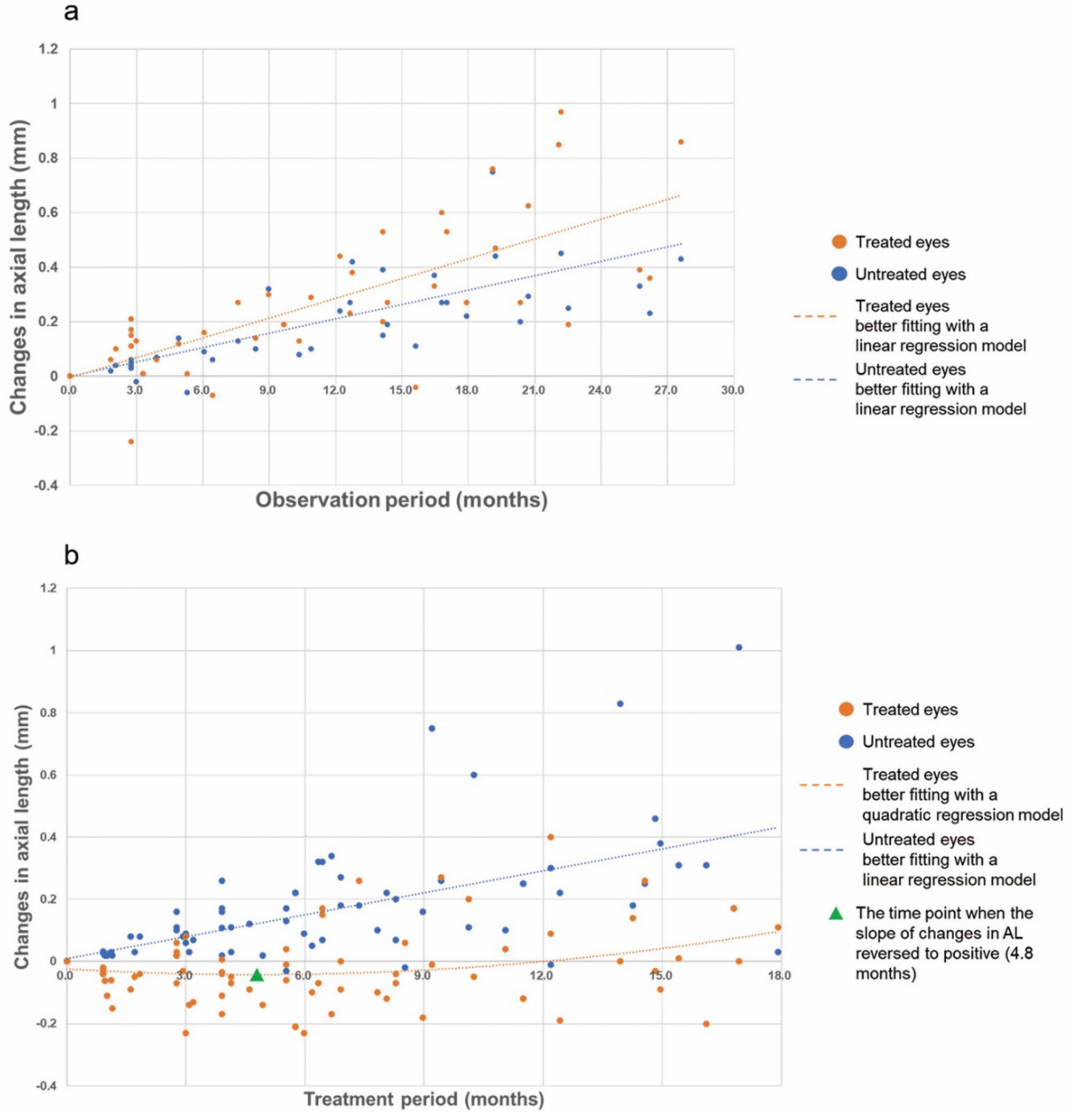
Best-fitting regression models to identify relationships between changes in AL and time in the treated and untreated eyes during the observation (*n* = 32) (**a**) and treatment (*n* = 40) (**b**) periods. (**a**) Best-fitting models of the changes in AL with time during the observation period in the treated and untreated eyes were both linear and produced high correlations (*R*^2^ = 0.647 and 0.639, respectively). The linear formulas are *y* = 0.0242*x* – 0.0038 for the treated eyes and *y* = 0.0176*x* – 0.0012 for the untreated eyes. (**b**) Best-fitting models of the changes in AL with time during the treatment period in the treated and untreated eyes were quadratic with a concave function (*R*^2^ = 0.068) and linear (*R*^2^ = 0.422), respectively. The green triangle indicates the turning point (*x* = 4.8 months) when the slope of the changes in AL became positive in the treated eyes. The quadratic formula is *y* = 0.0008*x*^2^ − 0.0076*x* − 0.0241 for the treated eyes and the linear formula is *y* = 0.0236*x* + 0.0085 for the untreated eyes.

The results of the regression analysis between AL (in mm) and time (in months) in the treated and untreated eyes during the observation period are shown in Fig. 2a. In both eyes, the best fit was obtained using linear regression models rather than quadratic regression models; that is, the adjusted *R*^2^ was larger for the linear regression models than the quadratic regression models. High correlations were obtained from the linear regression models (*R*^2^ = 0.647 in the treated eyes and 0.639 in the untreated eyes; the effect sizes are both considered to be large^13^).

The relationships between changes in AL (in mm) and time (in months) in the treated and untreated eyes during the treatment period are shown in Fig. 2b. In contrast to the data for the observation period, the best fit was obtained using the quadratic regression model with a concave function in the treated eyes; that is, the adjusted *R*^2^ was larger in the quadratic regression model than in the linear regression model. In the treated eyes, time (*x*) correlated with the changes in AL (*y*) in the quadratic model, in which a negative correlation was observed from 0 to 4.8 months and a positive correlation from 4.8 to 18 months (*R*^2^ = 0.068; the effect size is small to medium^13^). The turning point (the time *x* at which changes in AL *y* changed positively) was *x* = 4.8 months and *y* = − 0.042 mm. In the untreated eyes, time (*x*) correlated with the changes in AL (*y*) in the linear model (*R*^2^ = 0.422; the effect size is large^13^). The velocity of AL elongation is faster during the treatment period than during the observation period; the slopes were 0.0236 and 0.0176, respectively (Fig. 2).

## Discussion

To the best of our knowledge, this is the first study of the daily use of 0.125% atropine treatment for the eye with the longer AL in children with axial anisometropia. After treatment, AL was controlled in the treated eye but elongated significantly in the untreated eye (Fig. 1a and b). The difference between the two eyes decreased from 0.57 to 0.22 mm (*p* < 0.001) after the 1-year treatment (Fig. 1c). This finding suggests that daily use of 0.125% atropine in the eye with the longer AL may be an effective and rational therapy for pediatric axial anisometropia. However, in the current study, the differences in AL and SEQ between the two eyes remained significantly different after a mean treatment time of 13.7 months (Table 1). Fortunately, this AL difference decreased with treatment time (Figs. 1c and 2b). It is possible that more treatment time may ultimately cure anisometropia, although this needs to be confirmed in further investigation.

In the current study, we observed that the eye with the longer AL elongated faster than the other eye during the observation period. We estimate that if the eyes were left untreated, the difference in AL between eyes would have increased further, which would have resulted in worsening of anisometropia (Table 1 and Fig. 2a). This phenomenon has been noted in previous studies indirectly^4^. These findings suggest that retarding the elongation of AL in the eye with longer AL is necessary.

Previous studies have indicated that AL elongation can be retarded by lower-dose atropine treatment in both eyes^5,8^ and that lower-dose atropine had fewer visual side effects and less chance of a rebound effect compared with higher-dose atropine^10^. Therefore, we used the lowest dose of atropine (0.125%) covered by public health insurance in Taiwan in the current study. We found that AL was controlled and even shortened within 4.8 months of treatment in the treated eye. This observation is consistent with that of previous studies, although the mechanism remains unclear^5,14,15^. However, since we cannot exclude data for the first 4.8 months given the retrospective nature of our study, we acknowledge that we may have overestimated the AL-retarding effect of atropine by using the outcome measure of the calculated annual changes in AL from short-term measurement of AL in some cases. Nevertheless, the average follow-up time was 13.7 (median 11.6) months, so the overestimation is supposed to be minimal.

The difference in speed of AL elongation between the two eyes during the observation period may have different explanations, one of which may be accommodation imbalance. By balancing the accommodative difference between the two eyes, spectacles, in particular specially designed spectacles such as defocus incorporated multiple segments (DIMS), may also have a role in anisometropia control, although further studies are needed^16–19^. Although the conditions of anisometropia of case 5 and 15 might have improved without treatment according to the trends in changes in AL during the observation period, their parents requested control of myopia in the longer-AL eye. Therefore, we agreed to treat them after providing a full explanation of the advantages and disadvantages to the parents. The differences in AL between their two eyes decreased faster after treatment. This finding suggests that anisometropia may be solved in a faster way by monocular atropine treatment even with the tendency to self-recovery.

The AL of the untreated eye during the treatment period elongated faster than that during the observation period (Fig. 2), which resulted in a decrease in the difference in interocular AL after monocular treatment with 0.125% atropine (Figs. 1c and 2). A possible explanation is that most of the near vision relied on the untreated eye because of the incapability of accommodation in the treated eye^20^. Brain adaptation to produce clear images may also be involved^20,21^. Previous studies have not provided data during the observation period^5^. Further investigation of these observations is warranted.

Anisometropia can be classified into five types: simple myopic, compound myopic, simple hyperopic, compound hyperopic, and mixed anisometropic^5,6^. Different treatments may be suitable for different types of anisometropia. For the most common type, compound myopic anisometropia, studies have shown that orthokeratology (ortho-k) is a safe and effective method for treating this type of anisometropia by retarding AL growth. Differences in the efficacy of this retarding effect may result from differences in the target power of the ortho-k lens between the two eyes^7,22,23^. However, the types can shift. For example, compound myopic anisometropia can originate from hyperopic anisometropia, change into mixed anisometropia and then to myopic anisometropia because of continuous AL elongation at different speeds between eyes with age (Table 1 and Fig. 2a). Therefore, it may be more reasonable to use the AL as a categorizing parameter and the changes in AL as the main outcome measure in further investigation. Future studies are also needed to determine whether early intervention is an effective treatment for hyperopic anisometropia.

Atropine has flexibility for treating all types of anisometropia because of its pharmaceutical effects in AL retardation in eyes^5,21^. The frequency and/or concentration of atropine can also be adjusted for individual patients, and such adjustment has been presumed to change the efficacy for AL retardation^8^. Moreover, the shorter AL (more hyperopic) eye in people with hyperopic anisometropia may often exhibit some amblyopia, and atropine treatment of the eye with the longer AL may both prevent the eye with the longer AL from developing myopia and cure the amblyopic tendency in the other eye (penalization)^21,28^. This treatment may also be effective in reducing hyperopic anisometropia. We could not address this issue because we excluded data for patients with amblyopia and patients with a high degree of hyperopia (> 0.75 D) in longer-AL eye in accordance with the current consensus for myopia treatment and study design^8^. The use of atropine in the treatment of pure hyperopic anisometropia remains controversial^24^. Further research on this issue with larger sample sizes is needed. The combination therapy of atropine and ortho-k, DIMS lens, myopia-control soft contact lens, or various types of spectacles may open a new era for anisometropia treatment according to the condition for each individual patient^25–27^. The effects of combination treatments merit further investigation.

The common side effects from atropine treatment include photophobia and near-vision problems. However, our patients all tolerated the 0.125% atropine, and none refused treatment because of adverse effects, possibly because of the use as monocular therapy with lower-dose atropine. These findings are consistent with those of a previous study^5^. The reason for the exclusion of 14 patients because their compliance was < 80% was that they forgot to use the eye drops before sleep.

The rebound effect after cessation of atropine should be evaluated in the future. A previous study^5^ found that all atropine-treated eyes exhibited faster myopia progression than the fellow untreated eyes after cessation of treatment, especially in the first half-year after stopping treatment. However, the authors provided data only for the changes in refractive error and did not report the changes in AL. After a careful literature review, we found a lack of data regarding the rebound effect on the changes in AL after cessation of monocular atropine treatment in children with anisometropia. At the end of the follow-up in the present study, nine of our patients were shifted to be treated with atropine in both eyes, five were still treated monocularly, five were lost to follow-up, and one was treated with ortho-k afterwards. Therefore, we were unable to collect data after the cessation of treatment or tapering from atropine. The changes in AL related to the rebound effect merit further investigation.

The current study has several limitations, including its small sample size and retrospective design. There was also a lack of some cycloplegic refractive error data at the time points of initiating and stopping treatments. This may have resulted in bias in the cycloplegic refractive error as an outcome measure. Therefore, we used the main outcome measure of the changes in AL, which was acquired by direct measurement with the same machine for each patient. The changes in AL may also be less affected by accommodation than cycloplegic refractive error for assessing axial anisometropia^9^. On the other hand, the real changes in the cycloplegic refractive error after monocular atropine treatment and the relationship between the changes in AL and SEQ merit future investigation. For the correlation between the changes in AL and time in the treated eyes during treatment period, the effect size is small to medium (*R*^2^ = 0.068, Fig. 2b)^13^, and thus, further large-scale studies to confirm this relationship are warranted. Moreover, our study design was also too specific to provide data regarding different concentrations or frequencies of atropine treatments between the two eyes. Future studies of this issue are necessary for identifying the effects of different concentrations and/or frequencies of atropine treatments on different types of anisometropia.

In conclusion, the current study found that the eye with the longer AL elongated faster than the other eye, which resulted in worsening anisometropia during the observation period. However, after daily use of monocular atropine treatment in the eye with the longer AL, the AL stabilized in the treated eye but elongated faster than in the observation period in the untreated eye. The difference in AL between the two eyes decreased rapidly during the treatment period. The results of this pilot study suggest that monocular 0.125% atropine treatment may be an effective treatment for axial anisometropia. Future larger studies of atropine treatment for axial anisometropia are needed to confirm these results.

## Supplementary Information

Supplementary Table 1.

Supplementary Table 2.
