# Supplementary material for: Evaluation of axial length to identify the effects of monocular 0.125% atropine treatment for pediatric anisometropia
Source: Sci Rep. 2021 Nov 2;11:21511. doi: 10.1038/s41598-021-96414-4 (PMC8563952; doi:10.1038/s41598-021-96414-4)
Supplement: Supplementary file 1 — Supplementary Table 1. [file 41598_2021_96414_MOESM1_ESM.docx]

**Supplementary Table 1.** Baseline characteristics of the patients at the first visit, before initiating monocular atropine treatment, and after treatment

| Case No. | Sex | Age^†^ (years) | Time points | Days after baseline (months) | Treated eye | | | | Untreated eye | | | |
| --- | --- | --- | --- | --- | --- | --- | --- | --- | --- | --- | --- | --- |
|  |  |  |  |  | SRE, D | Ast, D | SEQ,D | AL, mm | SRE, D | Ast, D | SEQ,D | AL, mm |
| 1 | M | 14 | Baseline  Before Tx  After Tx | 0 (0)  0 (0)  515 (16.9) | –1.00 | –0.25 | –1.13 | 24.98 | 2.00 | –0.25 | 1.88 | 23.69 |
|  |  |  |  |  | –1.00 | –0.25 | –1.13 | 24.98 | 2.00 | –0.25 | 1.88 | 23.69 |
|  |  |  |  |  | –1.00^‡^ | –0.25 | –1.13 | 24.98 | –0.53^‡^ | –0.25 | –0.65 | 24.70 |
| 2 | F | 10 | Baseline  Before Tx  After Tx | 0 (0)  0 (0)  1302 (42.8) | –0.50 | –0.75 | –0.88 | 24.83 | 2.50 | –2.75 | 1.13 | 24.12 |
|  |  |  |  |  | –0.50 | –0.75 | –0.88 | 24.83 | 2.50 | –2.75 | 1.13 | 24.12 |
|  |  |  |  |  | –3.28^‡^ | –0.75 | –3.65 | 25.94 | -0.08^‡^ | –2.75 | –1.45 | 25.15 |
| 3 | M | 11 | Baseline  Before Tx  After Tx | 0 (0)  91 (3.0)  896 (29.5) | –0.50 | –0.50 | –0.75 | 24.43 | 0.75 | –0.75 | 0.38 | 23.89 |
|  |  |  |  |  | –0.83^‡^ | –0.50 | –1.08 | 24.56 | 0.80^‡^ | –0.75 | 0.43 | 23.87 |
|  |  |  |  |  | –0.43^‡^ | –0.50 | –0.68 | 24.40 | –0.48^‡^ | –0.75 | –0.85 | 24.38 |
| 4 | M | 11 | Baseline  Before Tx  After Tx | 0 (0)  84 (2.8)  420 (13.8) | –1.50 | –0.75 | –1.88 | 23.75 | 0.50 | –0.75 | 0.13 | 23.09 |
|  |  |  |  |  | –2.25 | –0.75 | –2.63 | 23.96 | 0 | –0.50 | –0.25 | 23.14 |
|  |  |  |  |  | –2.13^‡^ | –0.75 | –2.50 | 24.00 | 0^a^ | –0.50 | –0.25 | 23.24 |
| 5 | F | 13 | Baseline  Before Tx  After Tx | 0 (0)  196 (6.4)  384 (12.6) | –0.25 | –0.25 | –0.38 | 23.49 | 3.75 | –0.75 | 3.38 | 22.06 |
|  |  |  |  |  | –0.09^‡^ | –0.25 | –0.22 | 23.42 | 3.60^‡^ | –0.75 | 3.23 | 22.12 |
|  |  |  |  |  | 0.18^‡^ | –0.25 | 0.05 | 23.32 | 3.48^‡^ | –0.75 | 3.10 | 22.17 |
| 6 | M | 11 | Baseline  Before Tx  After Tx | 0 (0)  84 (2.8)  322 (10.6) | –1.25 | –0.50 | –1.50 | 23.98 | 0.25 | –1.00 | –0.25 | 23.39 |
|  |  |  |  |  | –1.63^‡^ | –0.50 | –1.88 | 24.13 | 0.10^‡^ | –1.00 | –0.40 | 23.45 |
|  |  |  |  |  | –1.38^‡^ | –0.50 | –1.63 | 24.03 | –0.15^‡^ | –1.00 | –0.65 | 23.55 |
| 7 | M | 15 | Baseline  Before Tx  After Tx | 0 (0)  0 (0)  150 (4.9) | –0.50 | –0.50 | –0.75 | 24.61 | 0.50 | –1.25 | –0.13 | 24.40 |
|  |  |  |  |  | –0.50 | –0.50 | –0.75 | 24.61 | 0.50 | –1.25 | –0.13 | 24.40 |
|  |  |  |  |  | –0.15^‡^ | –0.50 | –0.40 | 24.47 | 0.45^‡^ | –1.25 | –0.18 | 24.42 |
| 8 | F | 7 | Baseline  Before Tx  After Tx | 0 (0)  430 (14.1)  626 (20.6) | 0.50 | –0.25 | 0.38 | 22.42 | 1.00 | –0.25 | 0.88 | 22.32 |
|  |  |  |  |  | –0.83^‡^ | –0.25 | –0.96 | 22.95 | 0.03^‡^ | –0.25 | –0.10 | 22.71 |
|  |  |  |  |  | –1.25^‡^ | –0.25 | –1.38 | 23.12 | –0.78^‡^ | –0.25 | –0.90 | 23.03 |
| 9 | M | 13 | Baseline  Before Tx  After Tx | 0 (0)  797 (26.2)  1342 (44.1) | –0.25 | –0.25 | –0.38 | 22.67 | 0.25 | –0.25 | 0.13 | 22.58 |
|  |  |  |  |  | –1.15^‡^ | –0.25 | –1.28 | 23.03 | –0.33^‡^ | –0.25 | –0.46 | 22.81 |
|  |  |  |  |  | –1.43^‡^ | –0.25 | –1.55 | 23.14 | –0.40^‡^ | –0.25 | –0.53 | 22.84 |
| 10 | F | 8 | Baseline  Before Tx  After Tx | 0 (0)  675 (22.2)  878 (28.9) | 1.00 | –1.00 | 0.50 | 22.81 | 1.50 | –1.50 | 0.75 | 22.69 |
|  |  |  |  |  | –1.43^‡^ | –1.00 | –1.93 | 23.78 | 0.38^‡^ | –1.50 | –0.38 | 23.14 |
|  |  |  |  |  | –1.00^‡^ | –1.00 | –1.50 | 23.61 | –0.48^‡^ | –1.50 | –1.23 | 23.48 |
| 11 | F | 6 | Baseline  Before Tx  After Tx | 0 (0)  585 (19.2)  956 (31.4) | 0.50 | –2.00 | –0.50 | 22.41 | 0.25 | –1.50 | –0.50 | 22.20 |
|  |  |  |  |  | –0.68^‡^ | –2.00 | –1.68 | 22.88 | –0.85^‡^ | –1.50 | –1.60 | 22.64 |
|  |  |  |  |  | –1.68^‡^ | –2.00 | –2.68 | 23.28 | –1.60^‡^ | –1.50 | –2.35 | 22.94 |
| 12 | F | 7 | Baseline  Before Tx  After Tx | 0 (0)  783 (25.7)  1493 (49.1) | 0.75 | –3.50 | –1.00 | 23.08 | 1.75 | –3.25 | 0.13 | 22.74 |
|  |  |  |  |  | –0.23^‡^ | –3.50 | –1.98 | 23.47 | 0.93^‡^ | –3.25 | –0.70 | 23.07 |
|  |  |  |  |  | –0.90^‡^ | –3.50 | –2.65 | 23.74 | 0.21^‡^ | –3.25 | –1.42 | 23.36 |
| 13 | M | 10 | Baseline  Before Tx  After Tx | 0 (0)  331 (10.9)  912 (30.0) | 1.25 | –2.50 | 0 | 24.15 | 2.00 | –3.25 | 0.38 | 24.12 |
|  |  |  |  |  | 1.25 | –2.50 | 0 | 24.44 | 2.00 | –3.50 | 0.25 | 24.22 |
|  |  |  |  |  | 0.75^‡^ | –2.50 | –0.50 | 24.35 | 0.75^‡^ | –3.50 | –1.00 | 24.67 |
| 14 | M | 9 | Baseline  Before Tx  After Tx | 0 (0)  672 (22.1)  882 (29.0) | 1.00 | –3.00 | –0.50 | 22.80 | 0.75 | –1.50 | 0 | 22.44 |
|  |  |  |  |  | –1.13^‡^ | –3.00 | –2.63 | 23.65 | –1.38^‡^ | –1.50 | –2.13 | 23.29 |
|  |  |  |  |  | –1.13^‡^ | –3.00 | –2.63 | 23.65 | –2.05^‡^ | –1.50 | –2.80 | 23.56 |
| 15 | F | 10 | Baseline  Before Tx  After Tx | 0 (0)  685 (22.5)  1154 (37.9) | 0.75 | –0.50 | 0.50 | 22.62 | 2.25 | –1.25 | 1.63 | 22.31 |
|  |  |  |  |  | 0.25 | –0.75 | –0.13 | 22.81 | 1.50 | –1.00 | 1.00 | 22.56 |
|  |  |  |  |  | 0.25^‡^ | –0.50 | 0 | 22.82 | 0.85^‡^ | –1.25 | 0.23 | 22.87 |
| 16 | M | 10 | Baseline  Before Tx  After Tx | 0 (0)  56 (1.8)  108 (3.6) | 0.50 | –1.50 | –0.25 | 23.20 | 1.75 | –2.25 | 0.63 | 22.86 |
|  |  |  |  |  | 0.35^‡^ | –1.50 | –0.40 | 23.26 | 1.70^‡^ | –2.25 | 0.58 | 22.88 |
|  |  |  |  |  | 0.48^‡^ | –1.50 | –0.28 | 23.21 | 1.63^‡^ | –2.25 | 0.50 | 22.91 |
| 17 | F | 8 | Baseline  Before Tx  After Tx | 0 (0)  840 (27.6)  1400 (46.0) | 3.25 | –1.25 | 2.63 | 21.41 | 4.50 | –2.25 | 3.38 | 21.21 |
|  |  |  |  |  | 1.10^‡^ | –1.25 | 0.48 | 22.27 | 3.43^‡^ | –2.25 | 2.31 | 21.64 |
|  |  |  |  |  | 1.35^‡^ | –1.25 | 0.73 | 22.17 | 2.35^‡^ | –2.25 | 1.23 | 22.07 |
| 18 | M | 14 | Baseline  Before Tx  After Tx | 0 (0)  0 (0)  140 (4.6) | –2.50 | –0.50 | –2.75 | 26.80 | –0.50 | –0.75 | –0.88 | 25.84 |
|  |  |  |  |  | –2.50 | –0.50 | –2.75 | 26.80 | –0.50 | –0.75 | –0.88 | 25.84 |
|  |  |  |  |  | –2.30^‡^ | –0.50 | –2.55 | 26.72 | –0.80^‡^ | –0.75 | –1.18 | 25.96 |
| 19 | F | 7 | Baseline  Before Tx  After Tx | 0 (0)  84 (2.8)  364 (12.0) | –0.50 | –0.25 | –0.63 | 23.73 | 0.50 | –0.50 | 0.25 | 23.48 |
|  |  |  |  |  | –0.78^‡^ | –0.25 | –0.91 | 23.84 | 0.23^‡^ | –0.50 | –0.02 | 23.59 |
|  |  |  |  |  | –0.75^‡^ | –0.25 | –0.88 | 23.83 | -1.65^‡^ | –0.50 | –1.90 | 24.34 |
| 20 | M | 9 | Baseline  Before Tx  After Tx | 0 (0)  84 (2.8)  535 (17.6) | –1.50 | –0.50 | –1.75 | 24.51 | 0.75 | –1.25 | 0.13 | 23.80 |
|  |  |  |  |  | –1.93^‡^ | –0.50 | –2.18 | 24.68 | 0.65^‡^ | –1.25 | 0.03 | 23.84 |
|  |  |  |  |  | –1.85^‡^ | –0.50 | –2.10 | 24.65 | -0.50^‡^ | –1.25 | –1.13 | 24.30 |

AL, axial length; Ast, astigmatism; D, diopter; F, female; M, male; SEQ, spherical equivalent refractive error; SRE, spherical refractive error; Tx, treatment.

^†^Age of first monocular treatment; ^‡^calculated from baseline cycloplegic refractive error and the changes of AL.
