# Supplementary material for: Evaluation of axial length to identify the effects of monocular 0.125% atropine treatment for pediatric anisometropia
Source: Sci Rep. 2021 Nov 2;11:21511. doi: 10.1038/s41598-021-96414-4 (PMC8563952; doi:10.1038/s41598-021-96414-4)
Supplement: Supplementary file 2 — Supplementary Table 2. [file 41598_2021_96414_MOESM2_ESM.docx]

**Supplementary Table 2.** Changes in AL during observation and treatment periods

| Case No. | Observation period | Treated eye | | Untreated eye | |
| --- | --- | --- | --- | --- | --- |
|  | Treatment period | Δ AL (mm) | Δ AL/annum | Δ AL (mm) | Δ AL/annum |
| 1 | 0 (0) | None | None | None | None |
|  | 515 (16.9) | 0 | 0 | 1.01 | 0.716 |
| 2 | 0 (0) | None | None | None | None |
|  | 1302 (42.8) | 1.11 | 0.311 | 1.03 | 0.289 |
| 3 | 91 (3.0) | 0.13 | 0.521 | –0.02 | –0.080 |
|  | 805 (26.5) | –0.16 | –0.073 | 0.51 | 0.231 |
| 4 | 84 (2.8) | 0.21 | 0.913 | 0.05 | 0.217 |
|  | 336 (11.1) | 0.04 | 0.043 | 0.10 | 0.109 |
| 5 | 196 (6.4) | –0.07 | –0.130 | 0.06 | 0.112 |
|  | 188 (6.2) | –0.10 | –0.194 | 0.05 | 0.097 |
| 6 | 84 (2.8) | 0.15 | 0.652 | 0.06 | 0.261 |
|  | 238 (7.8) | –0.10 | –0.153 | 0.10 | 0.153 |
| 7 | 0 (0) | None | None | None | None |
|  | 150 (4.9) | –0.14 | –0.341 | –0.02 | 0.049 |
| 8 | 430 (14.1) | 0.53 | 0.450 | 0.39 | 0.331 |
|  | 196 (6.4) | 0.17 | 0.317 | 0.32 | 0.596 |
| 9 | 797 (26.2) | 0.36 | 0.165 | 0.23 | 0.105 |
|  | 545 (17.9) | 0.11 | 0.074 | 0.03 | 0.020 |
| 10 | 675 (22.2) | 0.97 | 0.525 | 0.45 | 0.243 |
|  | 203 (6.7) | –0.17 | –0.306 | 0.34 | 0.612 |
| 11 | 585 (19.2) | 0.47 | 0.293 | 0.44 | 0.275 |
|  | 371 (12.2) | 0.40 | 0.394 | 0.30 | 0.295 |
| 12 | 783 (25.7) | 0.39 | 0.182 | 0.33 | 0.154 |
|  | 710 (23.3) | 0.27 | 0.139 | 0.29 | 0.149 |
| 13 | 331 (10.9) | 0.29 | 0.320 | 0.10 | 0.110 |
|  | 581 (19.1) | –0.09 | –0.057 | 0.45 | 0.283 |
| 14 | 672 (22.1) | 0.85 | 0.462 | 0.85 | 0.462 |
|  | 210 (6.9) | 0 | 0 | 0.27 | 0.470 |
| 15 | 685 (22.5) | 0.19 | 0.101 | 0.25 | 0.133 |
|  | 469 (15.4) | 0.01 | 0.008 | 0.31 | 0.241 |
| 16 | 56 (1.8) | 0.06 | 0.391 | 0.02 | 0.130 |
|  | 52 (1.7) | –0.05 | –0.351 | 0.03 | 0.211 |
| 17 | 840 (27.6) | 0.86 | 0.374 | 0.43 | 0.187 |
|  | 560 (18.4) | –0.10 | –0.065 | 0.43 | 0.280 |
| 18 | 0 (0) | None | None | None | None |
|  | 140 (4.6) | –0.08 | –0.209 | 0.12 | 0.313 |
| 19 | 84 (2.8) | 0.11 | 0.478 | 0.11 | 0.478 |
|  | 280 (9.2) | –0.01 | –0.013 | 0.75 | 0.978 |
| 20 | 84 (2.8) | 0.17 | 0.739 | 0.04 | 0.174 |
|  | 451 (14.8) | –0.03 | –0.024 | 0.46 | 0.373 |

AL, axial length; Δ, the change in

Observation and treatment periods are expressed as days (months).
